# 

**Published:** 1899-10-07

**Authors:** 


					''The standard of highest purity."?Lancet.' I n ' OctobekJTth, 1899. ^
WLi?RY's fHl! OAD?*ys&
COCOA . _
ABSOLUTELY PURE. 1  NO DRUGS OR ALKALIES USED.
ORWICH UBMiniMB
v ^ v*-A&cotr western Infirmary
? 880. Vol. XXVII. [*??$?) Saturday,
Oct. 7th, 1899.
[Snbsoriptwii^TBrms.J pRICE TWOPENCE.

				

